# Cigarette smoking, e-cigarette use, and hypertension: a population-based cross-sectional study

**DOI:** 10.1038/s41371-026-01171-4

**Published:** 2026-06-04

**Authors:** Yusuff Adebayo Adebisi, Massimo Volpe, Jacob George, Ahmed K. Shukri, Najim Z. Alshahrani, Davide Capodanno, Bader Ali Almustafa, Riccardo Polosa

**Affiliations:** 1 College of Social Sciences, University of Glasgow, 40 Bute Gardens, Glasgow, G12 8RT Scotland United Kingdom; 2 Department of Clinical and Experimental Medicine, University of Catania, Catania, Italy; 3 Hypertension & Cardiovascular Prevention Unit, IRCCS San Raffaele Roma, Rome, Italy; 4 Department of Clinical and Molecular Medicine, Università di Roma Sapienza, Rome, Italy; 5 University of Dundee Medical School, Dundee, Scotland UK; 6 Department of Family and Community Medicine, Faculty of Medicine, University of Jeddah, Jeddah, Saudi Arabia; 7 Division of Cardiology, AOU Policlinico G. Rodolico-San Marco, University of Catania, Catania, Italy; 8 Secretary General, World Hypertension League, Riyadh, Saudi Arabia; 9 Head, Scientific Committee, Saudi Hypertension Management Society, Riyadh, Saudi Arabia; 10 Center of Excellence for the Acceleration of HArm Reduction (CoEHAR), University of Catania, Catania, Italy

**Keywords:** Risk factors, Hypertension

## Abstract

Cigarette smoking is an established cardiovascular risk factor, but the association between electronic cigarette (e-cigarette) use and hypertension remains unclear. We examined this association in a nationally representative adult population in Scotland using pooled cross-sectional data from 22,187 adults aged ≥16 years in the Scottish Health Survey (2017–2022). Participants were classified into five mutually exclusive groups: never users (n = 11,760), former smokers (n = 6,201), current cigarette smoking only (n = 2,747), current e-cigarette use only (n = 907), and current dual use (n = 572). Hypertension was defined as self-reported doctor-diagnosed high blood pressure, excluding pregnancy-related hypertension. Multivariable logistic regression estimated adjusted odds ratios (ORs) and 95% confidence intervals (CIs). Secondary analyses disaggregated current e-cigarette use only by smoking history, and propensity score matching was performed as a sensitivity analysis. Overall hypertension prevalence was 26.5%, ranging from 19.0% among current exclusive e-cigarette users to 33.5% among former smokers. Compared with never users, current e-cigarette use only was associated with lower odds of hypertension (OR 0.77, 95% CI 0.63–0.93), whereas former smokers had higher odds (OR 1.10, 95% CI 1.02–1.20). No significant associations were observed for current cigarette smoking only (OR 0.94, 95% CI 0.84–1.06) or current dual use (OR 0.95, 95% CI 0.75–1.20). The inverse association for exclusive e-cigarette use was confined to former smokers who had switched to vaping (OR 0.77, 95% CI 0.63–0.94). In propensity score-matched analyses, the inverse association for current exclusive e-cigarette use was attenuated and no longer statistically significant, suggesting that this finding may partly reflect healthy switcher bias and residual confounding rather than a protective effect.

## Introduction

Hypertension is the leading modifiable risk factor for cardiovascular morbidity and mortality worldwide, contributing to approximately 10.4 million deaths annually [1, 2]. In Scotland, an estimated one in three adults has been diagnosed with high blood pressure, imposing a substantial burden on the National Health Service and representing a major public health priority [3]. Identifying modifiable behavioural determinants of hypertension prevalence therefore remains an important objective for population health research.

Cigarette smoking is an established risk factor for cardiovascular disease, though its relationship with measured blood pressure has long been characterised as paradoxical [4–6]. While acute nicotine exposure elevates blood pressure through multiple mechanisms including sympathetic activation and catecholamine release [7, 8], numerous cross-sectional epidemiological studies have reported that current smokers have similar or even lower blood pressure levels compared with non-smokers [4, 9–12]. This paradox has been attributed to several mechanisms, including the lower body weight of smokers, selection bias, survival effects, more intensive clinical management of cardiovascular risk factors in smokers, and the vasodilatory properties of chronic carbon monoxide exposure [12]. Despite these complexities, the overall cardiovascular harm of cigarette smoking is beyond dispute.

Electronic cigarettes (e-cigarettes) have emerged as a rapidly growing alternative nicotine delivery system [13, 14]. In Great Britain, an estimated 5.4 million adults aged 16 years and over currently used an e-cigarette daily or occasionally in 2024, overtaking the number of current cigarette smokers for the first time [15]. E-cigarettes are frequently used as a smoking cessation aid, and the UK regulatory and public health environment has been broadly supportive of their harm-reduction potential as related to combustible cigarettes [16]. Nevertheless, the long-term cardiovascular safety of e-cigarettes remains unclear and currently represents an area of active investigation.

The relationship between e-cigarette use and hypertension is not well established. Experimental studies have demonstrated that acute e-cigarette use raises blood pressure and heart rate through nicotine-mediated sympathetic activation [17, 18]. However, the epidemiological evidence from population-based studies is mixed. Some cross-sectional analyses using US survey data have reported increased odds of hypertension among exclusive e-cigarette users [19–21], while others have found no significant association [22]. Importantly, studies examining smokers who switched to e-cigarettes have reported improvements in blood pressure control over follow-up periods of 6 to 12 months [23, 24], suggesting that the net cardiovascular effect may depend critically on whether e-cigarette use replaces or supplements combustible cigarette smoking [25]. A further limitation of the existing literature is that most population-based studies have classified cigarette smoking and e-cigarette use crudely, often treating e-cigarette use as a binary variable without distinguishing between exclusive e-cigarette users, dual users, and former smokers who no longer use any nicotine product. This classification approach can obscure important heterogeneity. For instance, a current exclusive e-cigarette user who is a former smoker has a different exposure history and potentially different cardiovascular risk profile compared with a never-smoker who currently vapes.

The present study aimed to investigate the association between current cigarette smoking and e-cigarette use status and prevalent hypertension using pooled data from the Scottish Health Survey (SHeS) for the years 2017 to 2022. We classified participants into five mutually exclusive groups based on their current cigarette smoking and current e-cigarette use, prioritising current exposure as the most relevant framework for a cross-sectional analysis of prevalent disease. In a secondary analysis, we further disaggregated current exclusive e-cigarette users by their smoking history to determine whether any observed association was driven by former smokers who had switched to vaping or by never-smokers who currently vaped.

## Methods

### Study design, data source, and study population

This study was a pooled cross-sectional analysis of adult participants in the Scottish Health Survey (SHeS). The SHeS is a nationally representative, repeated cross-sectional household survey designed to monitor the health of people living in private households in Scotland [13, 26]. It collects detailed information on sociodemographic characteristics, health conditions, smoking and e-cigarette use, alcohol consumption, and physical activity. All adults aged 16 years and over in responding households are eligible for interview.

We pooled five survey waves: 2017, 2018, 2019, 2021, and 2022. The original pooled dataset comprised 31,286 participants (5,300 from 2017; 6,790 from 2018; 6,881 from 2019; 6,157 from 2021; and 6,158 from 2022). Participants aged under 16 years were excluded using the derived age-group variable, removing 8,925 observations and leaving 22,361 adults. Participants with missing information on hypertension (n = 20) or on the main exposure variable (n = 154) were further excluded, yielding an analysis sample of 22,187 adults for descriptive analyses and crude prevalence estimates.

The choice to pool multiple waves was made to improve statistical power for relatively uncommon exposure subgroups, particularly current exclusive e-cigarette users and current dual users, while maintaining a nationally representative adult sample.

### Exposure

The primary exposure was current cigarette smoking and e-cigarette use status, derived from self-reported cigarette smoking status and e-cigarette use. Cigarette smoking status was based on a derived three-category variable distinguishing current cigarette smokers, ex-smokers, and never smokers. E-cigarette use was based on a derived variable categorising respondents as current users, past users, or never users of e-cigarettes or vaping devices.

For the main analysis, participants were classified into five mutually exclusive groups based on their current cigarette smoking and e-cigarette use. Never users had never smoked cigarettes and did not currently use e-cigarettes. Former smokers had previously smoked cigarettes but did not currently smoke or use e-cigarettes. Current cigarette smoking only comprised participants who currently smoked cigarettes but did not currently use e-cigarettes. Current e-cigarette use only comprised participants who currently used e-cigarettes but did not currently smoke cigarettes; this group included both never smokers and former smokers. Current dual use comprised participants who currently used both cigarettes and e-cigarettes. Participants who had used e-cigarettes in the past but were not current users were classified according to their current smoking status (as never users, former smokers, or current cigarette smokers), because this classification prioritised current rather than historical exposure.

This approach was considered the most relevant exposure framework for a cross-sectional analysis of prevalent hypertension, as current behaviour is more directly related to present-day cardiovascular risk than past experimentation with e-cigarettes.

In a secondary analysis, we further disaggregated the current e-cigarette use only category by smoking history, yielding exclusive vapers who were never smokers, and exclusive vapers who were former smokers. This secondary classification was used to determine whether any observed association for current exclusive e-cigarette use was driven primarily by former smokers who had switched to vaping rather than by never smokers who currently vaped.

### Outcome

The outcome was hypertension, defined using the derived SHeS variable for doctor-diagnosed high blood pressure excluding pregnancy. Respondents were classified as having hypertension if they answered affirmatively and as not having hypertension otherwise. Participants with non-substantive responses were excluded. This variable was selected in preference to the broader “ever had high blood pressure” item because it more closely reflects a clinically relevant diagnosis and explicitly excludes pregnancy-related hypertension.

### Covariates

Covariates were selected a priori as plausible confounders of the association between the exposure and hypertension prevalence [9–11, 27]. Age was included using a seven-category derived variable (16–24, 25–34, 35–44, 45–54, 55–64, 65–74, and 75 years or older). Sex was included as male or female. Survey year was included to account for differences across waves in both smoking and e-cigarette use patterns and background prevalence of diagnosed hypertension. Area deprivation was measured using the Scottish Index of Multiple Deprivation (SIMD) quintile variable, ranging from least to most deprived. Educational attainment was based on highest qualification and retained as six categories: degree or higher, HNC/HND or equivalent, Higher grade or equivalent, Standard grade or equivalent, other school-level qualification, and no qualifications. Ethnicity was included using a five-group derived variable: White Scottish, White Other British, White Other, Asian, and Other minority ethnic.

We additionally adjusted for diabetes (self-reported, coded dichotomously), other cardiovascular disease (any cardiovascular condition excluding diabetes and high blood pressure, coded dichotomously), alcohol consumption (four categories: never drank, ex-drinker, drinks within guidelines, drinks beyond guidelines), and physical activity (four categories based on the Chief Medical Officer recommendations: meets recommendations, some activity, low activity, very low activity). Education and area deprivation were both retained because they capture distinct dimensions of socioeconomic position, namely individual educational attainment and neighbourhood-level deprivation. Body mass index was not included in the main model because self-reported height and weight data were available for only a minority of the pooled sample; including it would have resulted in substantial loss of observations and potential selection bias.

### Statistical analysis

Descriptive statistics were first used to summarise the characteristics of the study population across the five cigarette smoking and e-cigarette use groups. Categorical variables were presented as frequencies and percentages, and differences in their distribution across exposure groups were assessed using Pearson’s chi-squared test. Hypertension prevalence was then estimated within each group and compared using the chi-squared test.

The primary analysis examined the association between exposure group and prevalent reported diagnosis of hypertension using binary logistic regression. Odds ratios (ORs) and 95% confidence intervals (CIs) were estimated for both crude and adjusted models. In addition, adjusted predicted probabilities of hypertension and their 95% CIs were estimated from the fully adjusted model using marginal standardisation (margins) to facilitate interpretation of absolute differences in model-estimated prevalence across exposure groups. In the main regression analyses, the reference category was never users. The crude model included only the exposure variable. The fully adjusted model controlled for age group, sex, survey year, area deprivation, educational attainment, ethnicity, diabetes, other cardiovascular disease, alcohol use, and physical activity. These variables were selected a priori as plausible confounders because of their established associations with both smoking and e-cigarette use behaviour and cardiovascular health.

A secondary analysis disaggregated the current e-cigarette use only group by smoking history to distinguish exclusive e-cigarette users who had never smoked from those who were former smokers. The same covariate set and reference category (never users) were retained. This approach provided insight into whether any observed association was attributable to prior combustible cigarette exposure. Adjusted predicted probabilities were also estimated for this disaggregated model. In a supplementary analysis, the fully adjusted model was re-estimated with current cigarette smoking only as the reference category to directly compare hypertension odds between current smokers and current exclusive e-cigarette users.

Sensitivity analyses using propensity score matching were conducted for selected pairwise contrasts to assess the robustness of the regression findings to imbalance in measured covariates between exposure groups. Specifically, 1:1 nearest-neighbour propensity score matching without replacement was performed for current e-cigarette use only versus never users, current e-cigarette use only versus current cigarette smoking only, and current cigarette smoking only versus never users. Propensity scores were estimated using logistic regression including age, sex, survey year, area deprivation, educational attainment, ethnicity, diabetes, other cardiovascular disease, alcohol use, and physical activity. For each matched comparison, the average treatment effect on the treated (ATET) and 95% confidence interval were estimated, and post-matching covariate balance was assessed using standardised differences.

Because the outcome was prevalent hypertension measured cross-sectionally, all estimates are interpreted as associations rather than causal effects. Statistical significance was assessed using two-sided tests at the 0.05 level. All analyses were performed using Stata version 18.0 (StataCorp, College Station, TX, USA).

## Results

### Study population characteristics

Table 1 presents the characteristics of the 22,187 participants by exposure group. The largest group was never users (n = 11,760; 53.0%), followed by former smokers (n = 6,201; 27.9%), current cigarette smoking only (n = 2,747; 12.4%), current e-cigarette use only (n = 907; 4.1%), and current dual use (n = 572; 2.6%). All characteristics differed significantly across groups (p < 0.001 for each). Current exclusive e-cigarette users tended to be younger than former smokers and had a similar age profile to current smokers. Approximately 45% of current exclusive e-cigarette users were male. Current smokers and dual users were disproportionately concentrated in more deprived areas and had lower educational attainment compared with never users and former smokers. The sample was predominantly White Scottish across all groups, though this proportion was highest among current e-cigarette users (84.0%) and dual users (84.4%) and lowest among never users (74.2%), who had the greatest ethnic diversity. The prevalence of other cardiovascular disease was highest among former smokers (22.5%) and lowest among never users and current e-cigarette users (both 15.7%). Diabetes prevalence was lowest among current exclusive e-cigarette users (6.8%) and never users (6.9%) and highest among former smokers (10.0%). Current smokers and dual users were more likely to drink beyond guidelines and to be ex-drinkers compared with never users. Physical inactivity was most prevalent among current cigarette-only smokers (30.2% in the very low activity category) and lowest among never users (19.1%).

**Table 1:** Characteristics of the study population by cigarette smoking and e-cigarette use group (N = 22,187).

Characteristic	Never users (n = 11,760)	Former smokers (n = 6,201)	Current cigarette smoking only (n = 2,747)	Current e-cigarette use only (n = 907)	Current dual use (n = 572)	Total (N = 22,187)	p-value
**Age group, n (%)**							<0.001
16–24	1,053 (9.0)	144 (2.3)	194 (7.1)	63 (7.0)	53 (9.3)	1,507 (6.8)	
25–34	1,484 (12.6)	520 (8.4)	443 (16.1)	153 (16.9)	93 (16.3)	2,693 (12.1)	
35–44	1,645 (14.0)	734 (11.8)	441 (16.1)	158 (17.4)	110 (19.2)	3,088 (13.9)	
45–54	1,855 (15.8)	943 (15.2)	564 (20.5)	211 (23.3)	137 (24.0)	3,710 (16.7)	
55–64	2,219 (18.9)	1,231 (19.9)	543 (19.8)	188 (20.7)	108 (18.9)	4,289 (19.3)	
65–74	2,061 (17.5)	1,466 (23.6)	391 (14.2)	116 (12.8)	59 (10.3)	4,093 (18.5)	
75+	1,443 (12.3)	1,163 (18.8)	171 (6.2)	18 (2.0)	12 (2.1)	2,807 (12.7)	
**Sex, n (%)**							<0.001
Male	4,901 (41.7)	2,842 (45.8)	1,321 (48.1)	410 (45.2)	252 (44.1)	9,726 (43.8)	
Female	6,859 (58.3)	3,359 (54.2)	1,426 (51.9)	497 (54.8)	320 (55.9)	12,461 (56.2)	
**SIMD quintile, n (%)**							<0.001
Least deprived	2,856 (24.3)	1,297 (20.9)	262 (9.5)	117 (12.9)	51 (8.9)	4,583 (20.7)	
4^th^	2,946 (25.1)	1,483 (23.9)	403 (14.7)	137 (15.1)	94 (16.4)	5,063 (22.8)	
3^rd^	2,518 (21.4)	1,401 (22.6)	552 (20.1)	182 (20.1)	96 (16.8)	4,749 (21.4)	
2^nd^	1,997 (17.0)	1,150 (18.5)	686 (25.0)	245 (27.0)	151 (26.4)	4,229 (19.1)	
Most deprived	1,443 (12.3)	870 (14.0)	844 (30.7)	226 (24.9)	180 (31.5)	3,563 (16.1)	
**Education, n (%)**							<0.001
Degree or higher	5,208 (44.3)	2,312 (37.3)	457 (16.6)	228 (25.1)	109 (19.1)	8,314 (37.5)	
HNC/HND or equivalent	1,377 (11.7)	780 (12.6)	336 (12.2)	157 (17.3)	88 (15.4)	2,738 (12.3)	
Higher grade or equivalent	1,767 (15.0)	789 (12.7)	453 (16.5)	128 (14.1)	90 (15.7)	3,227 (14.5)	
Standard grade or equivalent	1,572 (13.4)	908 (14.6)	650 (23.7)	206 (22.7)	147 (25.7)	3,483 (15.7)	
Other school level	551 (4.7)	377 (6.1)	144 (5.2)	44 (4.9)	20 (3.5)	1,136 (5.1)	
No qualifications	1,285 (10.9)	1,035 (16.7)	707 (25.7)	144 (15.9)	118 (20.6)	3,289 (14.8)	
**Ethnicity, n (%)**							<0.001
White Scottish	8,726 (74.2)	4,745 (76.5)	2,256 (82.1)	762 (84.0)	483 (84.4)	16,972 (76.5)	
White Other British	1,794 (15.3)	1,013 (16.3)	272 (9.9)	98 (10.8)	56 (9.8)	3,233 (14.6)	
White Other	576 (4.9)	337 (5.4)	148 (5.4)	35 (3.9)	24 (4.2)	1,120 (5.0)	
Asian	414 (3.5)	55 (0.9)	32 (1.2)	3 (0.3)	4 (0.7)	508 (2.3)	
Other minority ethnic	250 (2.1)	51 (0.8)	39 (1.4)	9 (1.0)	5 (0.9)	354 (1.6)	
**Other CVD, n (%)**							<0.001
No	9,916 (84.3)	4,808 (77.5)	2,222 (80.9)	765 (84.3)	467 (81.6)	18,178 (81.9)	
Yes	1,844 (15.7)	1,393 (22.5)	525 (19.1)	142 (15.7)	105 (18.4)	4,009 (18.1)	
**Diabetes, n (%)**							<0.001
No	10,946 (93.1)	5,579 (90.0)	2,516 (91.6)	845 (93.2)	530 (92.7)	20,416 (92.0)	
Yes	814 (6.9)	622 (10.0)	231 (8.4)	62 (6.8)	42 (7.3)	1,771 (8.0)	
**Alcohol use, n (%)**							<0.001
Never drank	1,202 (10.2)	187 (3.0)	153 (5.6)	30 (3.3)	30 (5.2)	1,602 (7.2)	
Ex-drinker	919 (7.8)	757 (12.2)	404 (14.7)	108 (11.9)	72 (12.6)	2,260 (10.2)	
Drinks beyond guidelines	3,747 (31.9)	2,621 (42.3)	1,142 (41.6)	384 (42.3)	245 (42.8)	8,139 (36.7)	
Drinks within guidelines	5,706 (48.5)	2,571 (41.5)	988 (36.0)	366 (40.4)	217 (37.9)	9,848 (44.4)	
Missing	186 (1.6)	65 (1.0)	60 (2.2)	19 (2.1)	8 (1.4)	338 (1.5)	
**Physical activity, n (%)**							<0.001
Meets recommendations	7,801 (66.3)	3,850 (62.1)	1,513 (55.1)	555 (61.2)	325 (56.8)	14,044 (63.3)	
Some activity	1,197 (10.2)	674 (10.9)	274 (10.0)	107 (11.8)	60 (10.5)	2,312 (10.4)	
Low activity	465 (4.0)	236 (3.8)	109 (4.0)	40 (4.4)	31 (5.4)	881 (4.0)	
Very low activity	2,247 (19.1)	1,410 (22.7)	830 (30.2)	201 (22.2)	152 (26.6)	4,840 (21.8)	
Missing	50 (0.4)	31 (0.5)	21 (0.8)	4 (0.4)	4 (0.7)	110 (0.5)	

### Prevalence of hypertension

**Fig. 1:**
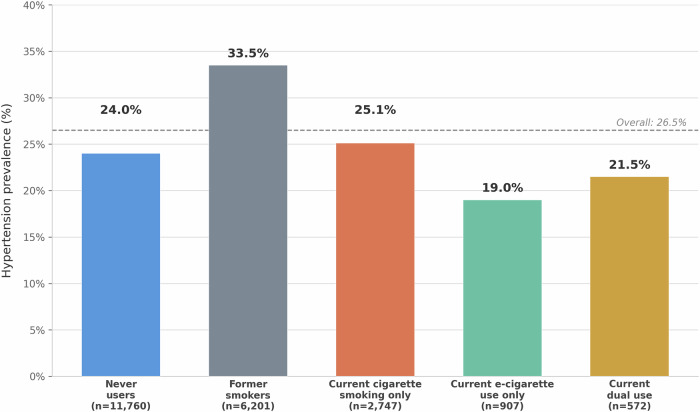
Prevalence of hypertension by cigarette smoking and e-cigarette use group (N = 22,187). Dashed line indicates overall prevalence (26.5%). P < 0.001 for overall comparison (Pearson χ² = 228.23).

Figure 1 shows the prevalence of reported diagnosis of hypertension by exposure group. Overall, 5,879 participants (26.5%) reported doctor-diagnosed hypertension. Prevalence varied substantially across groups (Pearson χ² = 228.23, p < 0.001). The highest prevalence was observed among former smokers (33.5%), followed by current cigarette smoking only (25.1%), never users (24.0%), current dual users (21.5%), and current exclusive e-cigarette users (19.0%).

### Main regression analysis

**Fig. 2:**
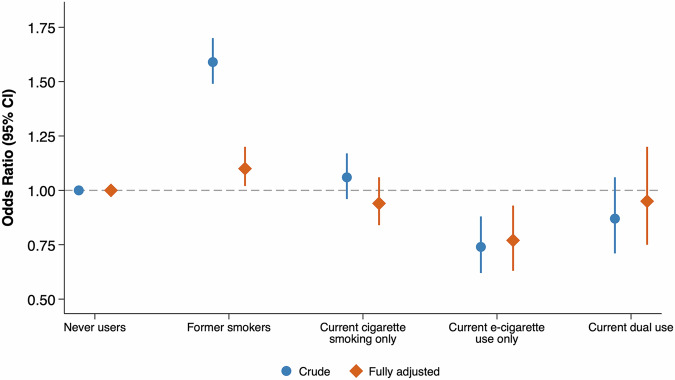
Crude and adjusted associations between cigarette smoking and e-cigarette use group and hypertension. Adjusted for age group, sex, area deprivation, educational attainment, ethnicity, other cardiovascular disease, diabetes, alcohol use, physical activity and survey year. Note: group sample sizes vary substantially (n = 11,760 for never users vs n = 907 for exclusive e-cigarette users and n = 572 for dual users); confidence intervals should be consulted when interpreting point estimates.

Figure 2 presents the crude and adjusted associations between exposure group and hypertension. In the crude analysis, former smokers had the highest odds of hypertension compared with never users (crude OR 1.59, 95% CI 1.49–1.70, p < 0.001), while current exclusive e-cigarette users had significantly lower crude odds (crude OR 0.74, 95% CI 0.62–0.88, p = 0.001). Current cigarette smoking only (crude OR 1.06, 95% CI 0.96–1.17, p = 0.248) and current dual use (crude OR 0.87, 95% CI 0.71–1.06, p = 0.172) were not significantly associated with hypertension in crude models.

After full adjustment for demographic, socioeconomic, clinical, and behavioural confounders, current exclusive e-cigarette use remained significantly associated with lower odds of hypertension (adjusted OR 0.77, 95% CI 0.63–0.93, p = 0.008). Former smokers retained a modest but statistically significant elevation in odds (adjusted OR 1.10, 95% CI 1.02–1.20, p = 0.013). The crude association for former smokers attenuated substantially after adjustment, indicating that age and other confounders largely explained the higher crude prevalence in this group. Current cigarette smoking only showed no significant association with hypertension after adjustment (adjusted OR 0.94, 95% CI 0.84–1.06, p = 0.325), consistent with the well-documented smoking–blood pressure paradox observed in cross-sectional data. Current dual use was also not significantly associated (adjusted OR 0.95, 95% CI 0.75–1.20, p = 0.664).

In adjusted margins analyses derived from the fully adjusted model, the predicted probability of hypertension was 26.3% (95% CI 25.6% to 27.0%) among never users, 27.8% (95% CI 26.9% to 28.7%) among former smokers, 25.4% (95% CI 23.9% to 26.9%) among current cigarette-only smokers, 22.5% (95% CI 19.9% to 25.1%) among current exclusive e-cigarette users, and 25.5% (95% CI 22.2% to 28.9%) among current dual users. These adjusted predicted probabilities mirror the regression findings, with the lowest model-estimated prevalence observed among exclusive e-cigarette users and little difference between cigarette-only smokers, dual users, and never users.

#### Secondary analysis by smoking history

Table 2 presents the secondary analysis in which current exclusive e-cigarette users were disaggregated by smoking history. Among the 907 exclusive e-cigarette users, 836 (92.2%) were former smokers and 71 (7.8%) were never smokers. Compared with never users, exclusive e-cigarette users who were former smokers had significantly lower adjusted odds of hypertension (adjusted OR 0.77, 95% CI 0.63 to 0.94; p = 0.010). Exclusive e-cigarette users who were never smokers showed a similar point estimate, but the association was not statistically significant and was estimated imprecisely (adjusted OR 0.74, 95% CI 0.34 to 1.62; p = 0.457), reflecting the small sample size in this subgroup. In adjusted margins analyses, the predicted probability of hypertension was 22.5% (95% CI 19.9% to 25.2%) among exclusive e-cigarette users who were former smokers and 22.1% (95% CI 11.6% to 32.6%) among exclusive e-cigarette users who were never smokers, compared with 26.3% (95% CI 25.6% to 27.0%) among never users. These findings suggest that the lower odds of hypertension observed among exclusive e-cigarette users in the main analysis were more clearly evident among those who were former smokers.

**Table 2:** Secondary analysis: current exclusive e-cigarette users disaggregated by smoking history.

Group	N	Adjusted OR (95% CI), p-value	Adjusted predicted probability, % (95% CI)
Never users	11,760	Ref	26.3 (25.6 to 27.0)
Exclusive e-cigarette users who were former smokers	836	0.77 (0.63 to 0.94), 0.010	22.5 (19.9 to 25.2)
Exclusive e-cigarette users who were never smokers	71	0.74 (0.34 to 1.62), 0.457	22.1 (11.6 to 32.6)

Never users served as the reference category. Adjusted for age group, sex, survey year, area deprivation, educational attainment, ethnicity, other cardiovascular disease, diabetes, alcohol use, and physical activity.

### Supplementary analysis

In a supplementary analysis, we re-estimated the fully adjusted model using current cigarette smoking only as the reference group to assess whether current exclusive e-cigarette use was associated with lower odds of hypertension relative to current smoking. Compared with current cigarette-only smokers, current exclusive e-cigarette users had lower adjusted odds of hypertension (OR 0.81, 95% CI 0.66–0.99, p = 0.045), whereas current dual users did not differ (OR 1.01, 95% CI 0.79–1.29, p = 0.957). Former smokers had higher odds than current cigarette-only smokers (OR 1.17, 95% CI 1.04–1.32, p = 0.010), and never users did not significantly differ (OR 1.06, 95% CI 0.94–1.19, p = 0.325). In adjusted margins analyses from the same model, the predicted probability of hypertension was 25.4% (95% CI 23.9%–26.9%) among current cigarette-only smokers, compared with 22.5% (95% CI 19.9%–25.1%) among current exclusive e-cigarette users, 25.5% (95% CI 22.2%–28.9%) among current dual users, 27.8% (95% CI 26.9%–28.7%) among former smokers, and 26.3% (95% CI 25.6%–27.0%) among never users.

### Sensitivity analyses

In propensity score–matched sensitivity analyses, the inverse association observed for current e-cigarette use only relative to never users in the main adjusted model was attenuated and no longer statistically significant (Table 3). In the fully adjusted logistic regression, current e-cigarette use only was associated with lower odds of hypertension than never users (OR 0.77, 95% CI 0.63 to 0.93; p = 0.008). However, after 1:1 propensity score matching, the average treatment effect on the treated (ATET) was −0.0247 (95% CI −0.0611 to 0.0117; p = 0.183), based on 907 current e-cigarette users matched to 907 never users.

**Table 3:** Sensitivity analyses comparing multivariable regression and propensity score–matched estimates for hypertension across selected nicotine-use contrasts.

Comparison	Eligible comparison sample, n	Matched treated, n	Matched controls, n	Adjusted OR (95% CI), p value	Matched ATET (95% CI), p value
Current e-cigarette use only vs never users	12,667	907	907	0.77 (0.63 to 0.93), p = 0.008	−0.0247 (−0.0611 to 0.0117), p = 0.183
Current e-cigarette use only vs current cigarette smoking only	3,654	907	907	0.81 (0.66 to 0.99), p = 0.045	−0.0364 (−0.0765 to 0.0038), p = 0.076
Current cigarette smoking only vs never users	14,507	2,747	2,747	0.94 (0.84 to 1.06), p = 0.325	−0.0059 (−0.0321 to 0.0202), p = 0.655

Adjusted odds ratios were obtained from multivariable logistic regression models adjusted for age group, sex, survey year, area deprivation, educational attainment, ethnicity, diabetes, other cardiovascular disease, alcohol use, and physical activity. Propensity score–matched estimates were obtained using 1:1 nearest-neighbour matching based on age, sex, survey year, area deprivation, educational attainment, ethnicity, diabetes, other cardiovascular disease, alcohol use, and physical activity. Negative ATET values indicate a lower matched prevalence of hypertension in the exposure group than in the reference group.

*ATET*, average treatment effect on the treated; *CI*, confidence interval; *OR*, odds ratio.

A similar pattern was observed when current e-cigarette use only was compared with current cigarette smoking only. In the supplementary adjusted model using current cigarette smoking only as the reference group, current e-cigarette use only was associated with lower odds of hypertension (OR 0.81, 95% CI 0.66 to 0.99; p = 0.045). In the corresponding propensity score–matched analysis, this association was attenuated and no longer statistically significant (ATET −0.0364, 95% CI −0.0765 to 0.0038; p = 0.076), based on 907 current e-cigarette users matched to 907 current cigarette smokers.

For current cigarette smoking only versus never users, findings were null in both the adjusted regression and matched analyses. The adjusted odds ratio was 0.94 (95% CI 0.84 to 1.06; p = 0.325), and the matched estimate was similarly close to the null (ATET −0.0059, 95% CI −0.0321 to 0.0202; p = 0.655), based on 2,747 current cigarette smokers matched to 2,747 never users. Overall, the propensity score–matched analyses were directionally consistent with the main regression models, but the inverse associations for current e-cigarette use only were weakened and no longer statistically significant after matching.

## Discussion

In this pooled cross-sectional analysis of 22,187 adults from the Scottish Health Survey (2017–2022), current exclusive e-cigarette use was associated with lower odds of prevalent hypertension compared with never users after adjustment for demographic, socioeconomic, clinical, and behavioural confounders. However, as discussed below, this finding is most plausibly explained by healthy switcher bias, residual confounding by age, body mass index, and other unmeasured factors, and the variable and uncontrollable effects of prior tobacco exposure in the vaping group, rather than by any protective effect of e-cigarette use. The association was concentrated among former smokers who had switched to vaping, whereas former smokers who had ceased all nicotine use showed modestly elevated odds of hypertension. Current cigarette smoking only and current dual use showed no significant associations. In supplementary analyses using current cigarette smoking as the reference, exclusive e-cigarette use was associated with modestly lower odds of hypertension than current smoking, whereas dual use did not differ from cigarette-only smoking. Importantly, propensity score–matched sensitivity analyses showed that the inverse associations for current e-cigarette use only versus never users and versus current cigarette smoking only were directionally consistent with the regression estimates but attenuated and no longer statistically significant after matching on observed covariates. This attenuation supports the interpretation that residual confounding accounts for a substantial proportion of the associations observed in the conventionally adjusted models.

Our finding that current exclusive e-cigarette use was associated with lower odds of hypertension diverges from the majority of existing cross-sectional epidemiological evidence. Cross-sectional analyses using US and Korean survey data have reported increased odds of hypertension among exclusive e-cigarette users [19–21], although a prospective analysis of the All of Us Research Program found no significant association in the main analysis [22]. These discrepancies may reflect important differences in the composition of the exclusive e-cigarette user population across countries. In the United Kingdom, the majority of adult e-cigarette users are current or former smokers who use e-cigarettes as cessation aids, whereas in the United States and South Korea a larger proportion of e-cigarette users may include younger individuals who initiated vaping without a prior smoking history. Differences in adjustment for confounders, in the definition of the reference group, and in the ascertainment of hypertension may also contribute to the divergent findings. Notably, the one prospective study that employed a longitudinal design, and therefore provides stronger evidence on temporality than the cross-sectional analyses, did not confirm the positive associations reported in cross-sectional data.

In contrast, our results are broadly consistent with the clinical literature on smokers who switched to e-cigarettes. A prospective study by Polosa and colleagues found that hypertensive smokers who switched to e-cigarettes experienced significant reductions in both systolic and diastolic blood pressure over 12 months, along with improved blood pressure control [23]. The ECLAT randomised controlled trial similarly reported that smokers who reduced or quit combustible cigarettes by switching to e-cigarettes showed long-term reductions in systolic blood pressure, particularly among those with elevated baseline blood pressure [24]. The VESUVIUS trial demonstrated significant improvements in endothelial function and vascular stiffness within one month of switching from tobacco cigarettes to e-cigarettes [28]. A recent systematic review with GRADE assessment found that smoking cessation and, in more limited evidence, switching to exclusive e-cigarette use were associated with early improvements in pulse wave velocity, augmentation index, and flow-mediated dilation, changes that are biologically compatible with improved arterial compliance and reduced vascular tone, and thus may contribute to lower blood pressure, although overall certainty was low (moderate only for RCT evidence on flow-mediated dilation) [25]. These findings support the interpretation that the lower odds of hypertension observed in our study among exclusive e-cigarette users, who were overwhelmingly former smokers, may partly reflect the removal of combustible tobacco exposure, though the contribution of healthy switcher bias and residual confounding cannot be disentangled in cross-sectional data. Importantly, these clinical findings do not explain why former smokers who switched to vaping had lower odds than former smokers who quit all nicotine, a pattern more consistent with selection effects than with a direct pharmacological benefit of e-cigarettes.

The finding that exclusive e-cigarette users had lower adjusted odds of hypertension than never users is counterintuitive and warrants careful interpretation. Several factors may explain this observation. First, the exclusive e-cigarette group was substantially younger than the never user group, with only 2.0% aged 75 years or older compared with 12.3% among never users. Although age was adjusted for categorically, residual confounding by age remains possible given the strength of the age–hypertension association. Second, body mass index, a major determinant of hypertension, could not be included in the model due to the high proportion of missing data, and differences in adiposity between groups may have contributed to the observed association. Third, individuals who successfully transition from cigarettes to e-cigarettes may differ from the general non-smoking population in unmeasured ways, including greater health awareness, higher engagement with healthcare services, and more favourable lifestyle behaviours not fully captured by the available covariates. This healthy switcher bias is a well-recognised source of confounding in observational studies of smoking cessation aids. Fourth, the prevalence of diabetes and other cardiovascular disease was similar or lower among exclusive e-cigarette users compared with never users (6.8% vs 6.9% for diabetes; 15.7% vs 15.7% for other cardiovascular disease), suggesting a broadly comparable or even more favourable cardiometabolic profile in this group. Fifth, the effects of prior tobacco exposure on vascular stiffness and arterial remodelling in the vaping group are variable in magnitude and duration, and impossible to fully account for in a cross-sectional design. Sixth, because the outcome is based on doctor-diagnosed hypertension, differences in healthcare contact or detection rates across behavioural groups may also contribute to the observed pattern; individuals who engage with smoking cessation services, for example, may have more frequent clinical encounters and thus a higher probability of receiving a hypertension diagnosis. These sources of unmeasured and residual confounding mean that the lower odds observed among exclusive e-cigarette users should not be interpreted as a protective effect of vaping against hypertension.

Our secondary analysis reinforces this interpretation. Of the 907 current exclusive e-cigarette users in our sample, 836 (92.2%) were former smokers. The significant association with lower hypertension odds was concentrated in this former-smoker subgroup, while the never-smoker vaper subgroup was too small (n = 71) to yield a meaningful estimate. This composition is typical of UK e-cigarette user populations [29–31] and indicates that the overall finding for exclusive e-cigarette users is attributable to former smokers who switched to vaping rather than to never-smokers who took up e-cigarette use independently.

The finding that dual users, who vape but continue to smoke, showed no significant association with hypertension provides an important contrast with exclusive e-cigarette users. If e-cigarette use itself were associated with lower hypertension prevalence, dual users who also vape would be expected to show similarly lower odds. They did not. The lower odds were observed only among those who had completely ceased combustible cigarette smoking and switched to e-cigarettes. This pattern suggests that the association is linked to the absence of current cigarette smoking rather than to e-cigarette use per se. This is consistent with clinical studies reporting that blood pressure reductions following e-cigarette adoption were greater among those who achieved complete cessation than among those who continued smoking at reduced levels [23, 24, 28].

The observation that current cigarette smoking was not significantly associated with hypertension after adjustment is consistent with the well-documented smoking–blood pressure paradox in cross-sectional epidemiology. For example, analyses of the Health Survey for England [10], the China Kadoorie Biobank involving nearly half a million participants [4], and Korean national survey data [32] have each reported that current smokers had similar or lower blood pressure levels compared with non-smokers after adjustment for confounders. A recent comprehensive review of both observational and Mendelian randomisation evidence concluded that observational studies suggest a paradoxical association between smoking and hypertension, while Mendelian randomisation studies have not established a clear causal relationship [12]. Proposed explanations include the lower body mass index of smokers, survival and selection bias inherent in cross-sectional designs, and possible chronic vasodilatory effects of carbon monoxide exposure. Our findings add to this body of evidence and suggest that the paradox extends to the comparison between current smokers and never users in the Scottish population.

The modest but significant elevation in odds among former smokers likely reflects the accumulated cardiovascular burden of prior smoking exposure. Former smokers tend to be older and to have longer histories of tobacco use; even after adjustment for age and other confounders, residual effects of historical smoking on vascular remodelling and arterial stiffness may contribute to higher hypertension prevalence in this group [9, 33–36]. Notably, the crude association for former smokers was substantially stronger (crude OR 1.59), indicating that the higher unadjusted prevalence in this group was largely explained by age and other confounders rather than by smoking history alone. An additional factor that may explain why former smokers who ceased all nicotine use had higher odds of hypertension than those who switched to e-cigarettes is post-cessation weight gain. Smoking cessation is typically accompanied by a mean weight gain of 4–5 kg in the first year, driven in part by the removal of nicotine’s appetite-suppressing and metabolic effects, and even modest weight gain can elevate blood pressure [37, 38]. Continued nicotine delivery through e-cigarettes may attenuate this appetite rebound and limit post-cessation weight gain. Consistent with this hypothesis, Polosa and colleagues reported that hypertensive smokers who switched to e-cigarettes experienced only trivial weight gain alongside significant reductions in systolic and diastolic blood pressure and improved blood pressure control over 12 months [23]. Although this mechanism could not be tested directly in the present study because body mass index data were largely unavailable, it offers a plausible partial explanation for the divergent findings between the two former-smoker subgroups that does not require invoking a direct antihypertensive effect of e-cigarette use.

Our findings have implications for the ongoing debate around e-cigarettes and cardiovascular harm reduction, though they require careful contextualisation. If the lower odds among exclusive e-cigarette users were explained primarily by tobacco avoidance through smoking cessation, one would expect former smokers who ceased all nicotine use to show at least equally low odds. They did not: former smokers who quit entirely had modestly elevated odds, whereas former smokers who switched to vaping had lower odds. This discrepancy argues against a straightforward smoking-cessation explanation and instead points towards healthy switcher bias – that is, former smokers who successfully transitioned to e-cigarettes may represent a systematically healthier subgroup with unmeasured advantages in terms of health motivation, body weight, and healthcare engagement. Differences in post-cessation weight gain may also contribute: former smokers who quit all nicotine are susceptible to weight gain that can elevate blood pressure, whereas continued nicotine delivery via e-cigarettes may attenuate this effect [23]. The propensity score–matched sensitivity analyses lend further support to the confounding interpretation. After matching on observed covariates, the inverse associations for current e-cigarette use only versus both never users and current cigarette smokers were attenuated and no longer statistically significant. Although the matched estimates remained directionally consistent with the regression findings, the loss of statistical significance after balancing observed confounders suggests that unmeasured confounding – particularly by body mass index and health motivation – likely explains a substantial share of the associations detected in conventionally adjusted models. These findings should therefore not be interpreted as evidence that e-cigarette use is protective against hypertension, either in the general population or among former smokers specifically. The cross-sectional nature of the data, the potential for healthy switcher bias, the inability to adjust for body mass index, the variable and uncontrollable effects of prior tobacco exposure in the vaping groups, and the small number of never-smoker vapers all preclude such a conclusion. The finding that the only prospective study to date did not confirm the positive cross-sectional associations reported elsewhere suggests that the relationship between e-cigarette use and hypertension may be more nuanced than cross-sectional data alone can reveal [22].

This study has several strengths. First, it used a large, nationally representative sample pooled across five survey waves, providing sufficient statistical power to examine relatively uncommon smoking and e-cigarette use patterns including exclusive e-cigarette use and dual use. Second, the five-category current exposure classification is conceptually clean, placing every participant into a group defined by their current behaviour rather than by past exposures that may not reflect present-day cardiovascular risk. Third, we adjusted for a comprehensive set of confounders spanning demographic, socioeconomic, clinical, and behavioural domains. Fourth, the secondary analysis disaggregating exclusive e-cigarette users by smoking history provides important mechanistic insight that is rarely available in the existing literature. Fifth, the supplementary analysis using current cigarette smoking as the reference group directly addresses the harm-reduction question, providing evidence from multiple analytical perspectives. Sixth, propensity score–matched sensitivity analyses complemented the conventional regression approach by balancing observed covariates across exposure groups, providing a more robust assessment of whether the observed associations are likely to reflect genuine effects or residual confounding.

Several limitations must be acknowledged. First, the cross-sectional design precludes causal inference. We cannot determine whether e-cigarette use preceded or followed the development of hypertension, nor can we exclude the possibility that individuals who switched to e-cigarettes were systematically healthier or more health-conscious than those who continued smoking, introducing selection bias. Second, hypertension was ascertained by self-report of a doctor’s diagnosis, which may underestimate true prevalence due to undiagnosed cases or overestimate it if participants misunderstood or misrecollected what their doctor communicated. This measure also cannot distinguish between treated and untreated hypertension. Nonetheless, self-reported doctor-diagnosed hypertension may also reflect differences in healthcare contact or detection across behavioural groups. Despite these limitations, it has been widely used in population health surveys and is less susceptible to the white-coat and measurement variability issues that affect single-occasion blood pressure readings [39]. Third, body mass index was not included in the main model due to the high proportion of missing data for self-reported height and weight. Given the well-established association between body weight and both smoking behaviour and hypertension [37, 38, 40], unmeasured confounding by adiposity is a particularly important limitation for interpreting the lower odds observed among exclusive e-cigarette users. Fourth, the survey did not collect information on the intensity or duration of e-cigarette use, the type of device used, or the nicotine concentration of e-liquids. These factors could modify the association between e-cigarette use and cardiovascular outcomes. Fifth, the duration of smoking cessation prior to e-cigarette adoption was not available, and the cardiovascular benefits of cessation are known to accumulate over time. Sixth, because the SHeS is a repeated cross-sectional survey, some participants may have contributed to more than one survey wave, though the probability of this is low given the large sampling frame.

Future research should employ longitudinal designs to establish temporality between switching to e-cigarettes and changes in blood pressure. Studies that capture the timing of smoking cessation, the onset of e-cigarette use, and subsequent blood pressure trajectories would be particularly informative. Additionally, studies that can adjust for body mass index and include objective measures of blood pressure would strengthen the evidence base. The growing population of e-cigarette users in the UK and globally, with e-cigarette users now outnumbering cigarette smokers in Great Britain for the first time, underscores the urgency of understanding the long-term cardiovascular consequences of these products.

In conclusion, current exclusive e-cigarette use was associated with lower odds of prevalent hypertension compared with never users in conventionally adjusted models, an association concentrated among former smokers who had switched to vaping. However, these associations were attenuated and no longer statistically significant in propensity score–matched sensitivity analyses. Current cigarette smoking showed no significant association with hypertension in either analytical approach, consistent with the established cross-sectional smoking–blood pressure paradox, and dual use showed no association. The divergence between former smokers who switched to vaping and those who ceased all nicotine use may partly reflect differences in post-cessation weight gain, healthy switcher bias, and residual confounding by unmeasured factors including body mass index and health motivation. Taken together, the findings do not support a protective effect of e-cigarette use on hypertension. Prospective studies with objective blood pressure measurements, adjustment for body mass index, and detailed data on smoking duration and cessation timing are needed to establish causality.

## Summary

### What is known about this topic

- Cigarette smoking is a well-established cardiovascular risk factor, but population-based evidence on the association between e-cigarette use and hypertension remains inconsistent, particularly when exclusive e-cigarette use, dual use, and smoking history are not clearly distinguished.

### What this study adds

- In a nationally representative sample of Scottish adults, exclusive e-cigarette use was associated with lower odds of self-reported doctor-diagnosed hypertension in conventionally adjusted models.
- This inverse association was concentrated among former smokers who had switched to vaping, not among dual users, and estimates for never-smoker vapers were imprecise because that subgroup was small.
- Current cigarette-only smoking and current dual use were not significantly associated with hypertension after adjustment.
- Propensity score matching attenuated the inverse associations and rendered them non-significant, suggesting healthy switcher bias and residual confounding rather than a protective effect of e-cigarette use.

## Data Availability

This study used secondary data from the Scottish Health Survey. No new datasets were generated for this study. The Scottish Health Survey datasets are available to registered users through the UK Data Service, subject to the applicable data access and licence conditions. The authors do not have permission to redistribute the data. Researchers wishing to access the data should apply through the UK Data Service Scottish Health Survey series.
